# Global Identification of Genes Related to Nutrient Deficiency in Intervertebral Disc Cells in an Experimental Nutrient Deprivation Model

**DOI:** 10.1371/journal.pone.0058806

**Published:** 2013-03-08

**Authors:** Hideki Sudo, Katsuhisa Yamada, Koji Iwasaki, Hideaki Higashi, Manabu Ito, Akio Minami, Norimasa Iwasaki

**Affiliations:** 1 Department of Advanced Medicine for Spine and Spinal Cord Disorders, Hokkaido University Graduate School of Medicine, Sapporo, Japan; 2 Department of Orthopaedic Surgery, Hokkaido University Graduate School of Medicine, Sapporo, Japan; 3 Division of Infection and Immunity, Hokkaido University Research Center for Zoonosis Control, Sapporo, Japan; University of Jaén, Spain

## Abstract

**Background:**

Intervertebral disc degeneration is a significant cause of degenerative spinal diseases. Nucleus pulposus (NP) cells reportedly fail to survive in large degenerated discs with limited nutrient availability. Therefore, understanding the regulatory mechanism of the molecular response of NP cells to nutrient deprivation may reveal a new strategy to treat disc degeneration. This study aimed to identify genes related to nutrient deprivation in NP cells on a global scale in an experimental nutrient deprivation model.

**Methodology/Principal Findings:**

Rat NP cells were subjected to serum starvation. Global gene expression was profiled by microarray analysis. Confirmation of the selected genes was obtained by real-time polymerase chain reaction array analysis. Western blotting was used to confirm the expression of selected genes. Functional interactions between p21^Cip1^ and caspase 3 were examined. Finally, flow cytometric analyses of NP cells were performed. Microarray analysis revealed 2922 differentially expressed probe sets with ≥1.5-fold changes in expression. Serum starvation of NP cells significantly affected the expression of several genes involved in DNA damage checkpoints of the cell cycle, including *Atm*, *Brca1*, *Cdc25*, *Gadd45*, *Hus1*, *Ppm1D*, *Rad 9*, *Tp53*, and *Cyclin D1*. Both p27^Kip1^ and p53 protein expression was upregulated in serum-starved cells. p21^Cip1^ expression remained in NP cells transfected with short interfering RNA targeting caspase 3 (caspase 3 siRNA). Both G1 arrest and apoptosis induced by serum starvation were inhibited in cells transfected with caspase 3 siRNA.

**Conclusions/Significance:**

Nutrient deprivation in NP cells results in the activation of a signaling response including DNA damage checkpoint genes regulating the cell cycle. These results provide novel possibilities to improve the success of intervertebral disc regenerative techniques.

## Introduction

Intervertebral disc (IVD) degeneration is a significant cause of degenerative spinal diseases, such as spinal canal stenosis and IVD herniation, which have been usually treated by conventional surgical approaches. Although the etiology of IVD degeneration remains unclear, it is thought to be associated with genetic factors as well as excessive biomechanical loading [1]–[3]. Recent molecular biological approaches have also demonstrated that senescence or apoptosis in IVD cells may have an important role in IVD degeneration [4]–[8]. Deteriorated IVD cells decrease the capacity to synthesize proteoglycan, aggrecan, and type 2 collagen, leading to the dehydration of the nucleus pulposus (NP) with severe consequences for both the biology and biomechanical stability of the IVD [4].

Although some vascular supply is present at the ventral margin of the IVD, NP has an avascular structure encapsulated by an endplate and the annulus fibrosus [9]. Nutrients are supplied to the avascular NP cells by capillaries, which arise in the vertebral bodies, penetrate the subchondral plate, and terminate in loops at the boundary of the cartilaginous endplate [10]. The density of the capillary contacts at the endplate–bone junction is considered one of the major factors regulating nutrient supply to the discs [10]. Calcification of the endplate and a reduction in this pathway appear to lead to disc disruption [11]–[13]. It has been reported that NP cells may fail to survive with limited nutrient availability [7]. Thus, understanding the regulatory mechanism of the molecular response of NP cells to nutrient deprivation may lead to a new strategy to treat IVD degeneration. The aim of this study was to identify genes related to nutrient deprivation in NP cells on a global scale in an experimental nutrient deprivation model.

## Materials and Methods

### NP Cell Culture Conditions

All animal procedures in this study were specifically approved by the Institutional Animal Care and Use Committee (IACUC) at Hokkaido University (Permit Number: 08-0433). Lumbar IVDs from male Sprague-Dawley rats (age, 13 weeks) were harvested immediately after they were euthanized. The gel-like NP was separated from the annulus fibrosus using a dissecting microscope (Figure S1), and the tissue specimens were placed in a complete tissue culture medium consisting of Dulbecco’s modified Eagle’s medium (DMEM; SIGMA-ALDRICH, St. Louis, MO) supplemented with 10% fetal bovine serum (BioWhittaker Inc., Walkersville, MD), 1% penicillin/streptomycin, and 1.25 µg/mL Fungizone (Invitrogen, Carlsbad, CA). Specimens were centrifuged and treated with DMEM supplemented with 0.25% collagenase in a shaking incubator at 37°C for 30 min. After the cell suspension was filtered, primary cells were placed in 6-cm tissue culture dishes and incubated at 37°C in a humidified atmosphere of 5% CO_2_. When confluent, the cells were lifted using a 0.25% trypsin/EDTA (Invitrogen) solution and subcultured on 10-cm dishes [7].

Healthy intact human NP samples were obtained from 10 patients (one man and nine women, mean age±SD: 16.8±2.4 years) for polymerase chain reaction (PCR), western blot, and flow cytometric analyses. These patients had undergone spinal fusion for adolescent idiopathic scoliosis. NP cells were separated and cultured as described above. We obtained informed consent from the next of kin, caretakers, or guardians on the behalf of the minors (<20 years old) participants involved in this study and the consent was written and the documents were saved. The ethics committee of the Hokkaido University Graduate School of Medicine specifically approved this study.

### Experimental Protocol

To simulate nutrient deprivation in NP cells, we selected an *in vitro* serum starvation model [7], [8], [10], [14]. Rat or human NP cells were washed with phosphate-buffered saline (Invitrogen) followed by two washes with DMEM to remove any remaining culture medium and incubation in serum-deprived medium consisting of DMEM supplemented with 1% penicillin/streptomycin and 1.25 µg/mL Fungizone at 37°C with 5% CO_2_ and 20% O_2_. Because we previously reported that significant rat NP cell apoptosis occurred 48 h after serum starvation [7], the cells were harvested and analyzed at 48 h (6 or 48 h for western blot) after serum withdrawal. NP cells not subjected to serum starvation were used as untreated controls [7], [8].

### Microarray Analysis

Rat NP cells were serum-starved for 48 h. After total RNA was isolated from cultures using a FastPure™ RNA Kit (TaKaRa BIO, Otsu, Japan) and quantified spectrophotometrically, the suitability of its quality for use in microarray analysis was confirmed by analysis in the Agilent 2100 Bioanalyzer (Agilent Technologies, Palo Alto, CA). Gene expression analysis using the Whole Rat Genome Oligo DNA Microarray (Agilent Technologies) was performed using 500 ng of RNA in accordance with the manufacturer’s protocol (Quick Amp Labeling Kit, one-color, Agilent Technologies). Data from the scanned chips were normalized and analyzed using the Agilent Feature Extraction software (Agilent Technologies). Biological replicates of microarray analysis were performed three times.

### Gene Ontology (GO) and Kyoto Encyclopedia of Genes and Genomics (KEGG) Pathway Analysis

After probe sets were filtered using the criterion of a minimum 1.5-fold change in differential gene expression between the serum-starved and untreated control groups, the resulting list containing 2652 probes was used for functional categorization and pathway construction. Categorized lists were generated on the basis of *Rattus norvegicus* annotations for GO analysis (http://geneontology.org/) and KEGG pathway analysis (http://www.genome.ad.jp/kegg/) to obtain biological insight into the functional process and biological pathways. GO terms are divided into three families: biological process, cellular component, and molecular function. In this study, we used the total list of GO terms within the biological process categories. A probability was calculated to determine whether any GO terms or pathways annotate a specified list of genes at a frequency greater than that would be expected by chance. The probability was determined using Fisher’s exact test [15].

### Quantitative Real-time PCR (qRT-PCR) Analysis

qRT-PCR analysis was performed with RT^2^ Profiler PCR Arrays (Rat Cell Cycle, SABiosciences, Frederick, MD) to validate the rat microarray analysis according to the manufacturer’s protocols. One microgram of each total RNA was reverse-transcribed into cDNA using an RT^2^ First Strand Kit (SABiosciences), and qRT-PCR was performed using RT^2^ SYBR Green qPCR Master Mix (SABiosciences). For the reactions, a thermal cycler was programmed as follows: 95°C for 10 min; 40 cycles of 95°C for 15 s, 55°C for 40 s, and 72°C for 30 s. Expression data were analyzed using the SABiosciences expression analysis template. Two housekeeping genes (*Rplp1* and *Actb*) were used for each gene expression calculation, and the extent of change in the expression of each gene was calculated by the ΔC_t_ method. When ΔC_t_ was over 12 and therefore expression was thought to be extremely low, the gene was omitted from analysis [16].

qRT-PCR analysis of p53 and caspase 3 for rat samples were also performed. Total RNA extraction from NP cells incubated in 6-well plates (2.0×10^5^ cells/well) was performed using the NucleoSpin RNA II Kit. RNA was reverse-transcribed into cDNA, and real-time PCR was performed using a SYBR PrimeScript RT reagent Kit (TaKaRa BIO). For the real-time reactions, a thermal cycler was programmed as follows: 95°C for 30 s; 40 cycles of 95°C for 5 s, 60°C for 30 s, and 95°C for 1 min. Primers for p53, caspase 3, and glyceraldehyde phosphate dehydrogenase (GAPDH) were custom-designed and synthesized by TaKaRa BIO Inc. Primers for the rat samples were as follows: for p53, 5′ TGCAGTCAGGGACAGCCAAG 3′ and 5′ GAGGTGACCCACAACTGCACA 3′; for caspase 3, 5′-GAGACAGACAGTGGAACTGACGATG-3′and 5′-GGCGCAAAGTGACTGGATGA-3′; and for GAPDH, 5′-GACAACTTTGGCATCGTGGA-3′and 5′-ATGCAGGGATGATGTTCTGC-3′. qRT-PCR analysis of caspase 3 for human samples were also performed. Primers were as follows: for caspase 3, 5′-ACAGAACTGGACTGTGGCATTGAG-3′and 5′-GGCACAAAGCGACTGGATGA-3′; and for GAPDH, 5′-GCACCGTCAAGGCTGAGAAC-3′and 5′-TGGTGAAGACGCCAGTGGA-3′. The relative messenger RNA (mRNA) expression of target genes per GAPDH was calculated.

### Western Blot Analysis

Rat or human NP cells were serum-starved for 6 or 48 h. Cells were then lysed with 0.4 mL of ice-cold T-PER tissue protein extraction reagent (Pierce Biotechnology, Rockford, IL). Cell lysates (20 µg protein/lane) were loaded and separated on a 4–12% gradient polyacrylamide gel and transferred to polyvinylidene difluoride membranes by electroblotting. After blocking with 5% nonfat milk containing 0.3% Tween 20 (Bio-Rad Laboratories, Hercules, CA) for 1 h, the membranes were incubated overnight with antibodies to p15^Ink4b^ (Abcam, Cambridge, UK), p16^Ink4a^ (Abcam), p21^Cip1^ (Santa Cruz, CA), p27^Kip1^ (Santa Cruz), p53 (Abcam), and caspase 3 (Cell Signaling Technologies, MA) at 4°C. The membranes were washed three times with Tris-buffered saline-Tween 20 and further incubated with horseradish peroxidase-conjugated anti-rabbit IgG (Cell Signaling Technology) secondary antibody for 1 h. The membrane was then exposed to an enhanced chemiluminescent system, and a charge-coupled device image analyzer was used to visualize immunoreactive bands. β-actin was used as an internal control to confirm equal protein loading.

### Preparation and Transfection of Short Interfering RNA (siRNA)

siRNAs for rat p53 (p53 siRNA) and caspase 3 (caspase 3 siRNA) oligonucleotide were constructed as follows: for p53siRNA, sequence 1∶5′-CAAUUUCCCUCAAUAAGCUTT-3′and 5′-AGCUUAUUGAGGGAAAUUGTT-3′; sequence 2∶5′-CCACUAUCCACUACAAGUATT-3′and 5′-UACUUGUAGUGGAUAGUGGTT-3′; for caspase 3, sequence 1∶5′-GCACAUCCUCACUCGUGUUTT-3′and 5′-AACACGAGUGAGGAUGUGCTT-3′; sequence 2∶5′-GAAAGCCGAAACUCUUCAUTT-3′ and 5′-AUGAAGAGUUUCGGCUUUCTT-3′. siRNA for human caspase 3 were also constructed as follows: sequence 1∶5′-GCAUAUCAGUUGAGCUUCATT-3′and 5′-UGAAGCUCAACUGAUAUGCTT-3′; sequence 2∶5′GUAGAAGAGUUUCGUGAGUTT-3′ and 5′-ACUCACGAAACUCUUCUACTT-3′. A scrambled negative control siRNA without specific functions was also synthesized (sequences, 5′-UCUUAAUCGCGUAUAAGGCTT-3′and 5′-GCCUUAUACGCGAUUAAGATT-3′). Transfection was performed with 150 pmol of double-stranded siRNA premixed with Lipofectamine RNAiMax (Invitrogen) in Opti-MEM (Invitrogen) on 6-well tissue culture plates (2×10^5^ cells/well) according to the manufacturer’s instructions. Forty-eight hours after transfection, cells were serum-deprived for 48 h and harvested.

### Immunofluorescence

To confirm transduction of caspase 3siRNA in the rat NP cells, NP cells were visualized by fluorescence microscopy. Cells were seeded on 24-well plates (2×10^4^ cells/well), fixed for 10 min in 1% PBS-buffered paraformaldehyde, treated with 0.5% Triton X-100-PBS for 30 min, blocked with 1% BSA for 60 min, and incubated with primary antisera for caspase 3 (1∶100 dilution) (Thermo Scientific, Japan) for overnight in 1% BSA in PBS followed with secondary antisera for 30 min. Secondary sera used were FITC-conjugated Goad anti-Rabbit IgG (1∶100 dilution) (Jackson ImmunoResearch, PA).

### Analysis of Apoptosis and Cell Cycle

An FITC Annexin V Apoptosis Detection Kit (BD Biosciences, CA) was used in the analysis of apoptosis as previously described [7], [8]. Briefly, cells (3.6×10^5^) from each treatment group were incubated in 6-cm tissue culture dish, isolated, and centrifuged. Cell pellets were resuspended and incubated in 100 µ1 binding buffer containing annexin V-fluorescein isothiocyanate (FITC) and propidium iodide (PI). Both early apoptotic cells (FITC+/PI −) and late apoptotic cells (FITC+/PI +) were monitored with a flow cytometer (Coulter Epics XL Flow Cytometer; Beckman Coulter, CA). Likewise, PI staining method was used for detecting the cell cycle status using the manufacturer’s protocol. DNA was stained with 50 µ1 PI. Samples were kept for 1 hour in the dark at room temperature and DNA index was then measured by cytofluorimetric analysis.

### Statistical Analysis

All values in the text and figures were expressed as mean ± standard deviation. Experiments were performed three times per treatment group. Statistical analyses were performed with Student’s *t*-test unless otherwise noted. *P* values less than 0.05 were considered significant.

## Results

### Assessment of Microarray Gene Expression

Firstly, to identify the global molecular response to nutrient deprivation in rat NP cells, gene expression analysis was performed using microarrays. When categorized by biological process with GO analysis, several categories were upregulated including *regulation of physiological process*, *response to environmental stimulus*, *response to extracellular stimulus*, and *response to nutrient levels*. Downregulated GO terms included *anatomical structure development*, *organ development*, and *anatomical structure morphogenesis*. Genes connected with *regulation of programmed cell death* and *regulation of cell proliferation* were also activated or repressed by serum starvation (**Tables S1, S2**). Furthermore, we identified upregulated genes related to *response to nutrient levels* through GO analysis (**Table S3**).

The GO database does not cover every aspect of biology relevant to gene products, and many GO classes are overlapping or redundant. The KEGG pathways are compiled from multiple literature sources, and they integrate individual components into a unified pathway. Therefore, the KEGG pathway database was used to further characterize the enrichment of specific pathway components into functionally regulated gene groups [17]. Seventeen KEGG pathways were significantly enriched in genes associated with serum starvation (*P*<0.01) (**Table 1**). Of the significant pathways with low *P* values and biological significance, the category *pathways in cancer* was selected for further analysis.

**Table 1:** Highly significant (*P*<0.01) pathways based on KEGG database*.

Pathway (total gene count)	*P*-value	Count
*Up-regulate*		
Pathways in cancer (313)	0.00004	27
Fatty acid metabolism (41)	0.00012	8
Adipocytokine signaling pathway (64)	0.00061	9
Complement and coagulation cascades (67)	0.00087	9
Small cell lung cancer (92)	0.00237	10
B cell receptor signaling pathway (67)	0.00357	8
Tyrosine metabolism (29)	0.00424	5
PPAR signaling pathway (70)	0.00470	8
Toll-like receptor signaling pathway (90)	0.00675	9
T cell receptor signaling pathway (95)	0.00955	9
*Down-regulated*		
O-Glycan biosynthesis (6)	0.00034	6
Focal adhesion (185)	0.00058	18
Calcium signaling pathway (191)	0.00085	18
Glutathione metabolism (42)	0.00150	7
ECM-receptor interaction (73)	0.00297	9
Nitrogen metabolism (16)	0.00355	4
p53 signaling pathway	0.00357	9

* KEGG, Kyoto Encyclopedia of Genes and Genomes. “total gene count” means the number of genes in each pathway, which have already been registered in KEGG system, and “count” means the number of genes, which were expressed significantly in each pathway in this study.

### Validation Analysis of Gene Expression by qRT-PCR Array

Based on the differential pattern within the KEGG pathway, we further investigated genes associated with the cell cycle because this category is closely related to the categories of both cell proliferation and apoptosis in the KEGG pathway. To validate and determine the effects of serum starvation on cell cycle-related genes, pathway-specific qRT-PCR array analysis was performed on all samples used for microarray hybridization. The Rat Cell Cycle RT^2^ Profiler™ PCR Array profiles the expression of 84 genes critical to cell cycle regulation. This array contains genes that both positively and negatively regulate the cell cycle, the transitions between the phases, DNA replication, checkpoints, and arrest. Only genes that were significantly regulated at least 1.5-fold in the qRT-PCR array analysis were included in **Table 2**. The pattern of relative gene expression measured by qRT-PCR agreed with the microarray results. Serum starvation of NP cells significantly affected the expression of several genes, including the upregulation of ataxia telangiectasia mutated homolog (*Atm*), breast cancer 1 (*Brca1*), cell division cycle 25 (*Cdc25*), growth arrest and DNA-damage-inducible 45 (*Gadd45*), HUS1 checkpoint homolog (*Hus1*), protein phosphatase 1D (*Ppm1D*), cell cycle checkpoint protein RAD 9,21 (*Rad 9*,*21*), and tumor suppressor protein *Tp53*; and the downregulation of cyclin D1 (*Ccnd 1*) These genes are known to be DNA damage checkpoint genes in cell cycle.

**Table 2:** Genes with significant expression levels in serum-starved nucleus pulposus cells Only genes whose expression was significantly (*P*<0.05) up- or down-regulated at least 1.5 fold are shown.

GeneSymbol	Description	qRT-PCR		Array
		Fold change	*P* value	Fold change
Abl1	C-abl oncogene 1, receptor tyrosine kinase	1.78	0.033	1.28
Atm	Ataxia telangiectasia mutated homolog (human)	1.86	0.015	1.43
Brca1	Breast cancer 1	1.57	0.020	1.69
Ccnd1	Cyclin D1	−1.51	0.024	−1.59
Cdc25a	Cell division cycle 25 homolog A (S. pombe)	1.68	0.016	1.31
Gadd45a	Growth arrest and DNA-damage-inducible, alpha	1.82	0.038	1.58
Hus1	HUS1 checkpoint homolog (S. pombe)	1.74	0.042	1.46
Bcl2	B-cell CLL/lymphoma 2	1.89	0.003	1.82
Rad21	RAD21 homolog (S. pombe)	1.62	0.003	1.49
Msh2	MutS homolog 2 (E. coli)	1.63	0.003	1.23
Notch2	Notch homolog 2 (Drosophila)	1.56	0.006	1.38
Ppm1d	Protein phosphatase 1D magnesium-dependent, delta isoform	1.74	0.039	1.39
Rad9	RAD9 homolog (S. pombe)	1.64	0.013	1.37
Stag1	Stromal antigen 1	1.52	0.044	1.52
Terf1	Telomeric repeat binding factor (NIMA-interacting) 1	1.56	0.007	1.24
Tfdp2	Transcription factor Dp-2 (E2F dimerization partner 2)	1.78	0.020	1.39
Tp53	Tumor suppressor protein p53	2.07	0.010	1.403

### Western Blot Findings

**Figure 1:**
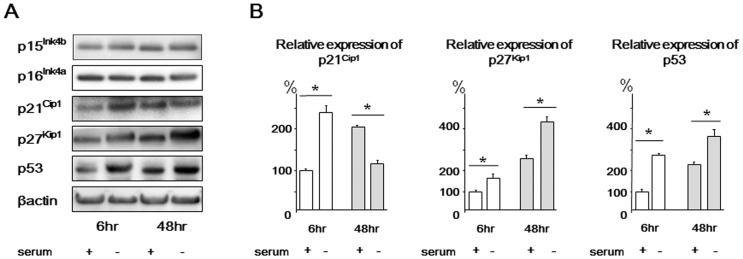
Western blots of p15^Ink4b^, p16^Ink4a^, p21^Cip1^, p27^Kip1^, and p53 in rat nucleus pulposus cells. Cells were harvested after 6 or 48 h of serum starvation. Cells not subjected to serum starvation were used as untreated controls. β-actin was used as an internal control. (A) Representative western blot analysis. (B) Densitometry analyses were performed to quantify the levels of p21^Cip1^, p27^Kip1^, and p53 via normalization to beta-actin. Results are representative of three independent experiments. Values are expressed as the mean ± SD (* = P<0.05).

Based upon both microarray and qRT-PCR array analysis, we further examined the protein levels of selected genes downstream of the aforementioned DNA damage checkpoint genes. Western blot analysis using rat NP cells demonstrated that p21^Cip1^ protein expression was increased after 6 h and decreased after 48 h of serum starvation. Both p27^Kip1^ and p53 protein expression was increased after both 6 and 48 h of serum starvation. Conversely, p15^Ink4b^ and p16^Ink4a^ protein expression was unchanged even after 48 h of serum starvation (**Figure 1**). These cells displayed the same expression trends as human NP cells (**Figure S2**).

**Figure 2:**
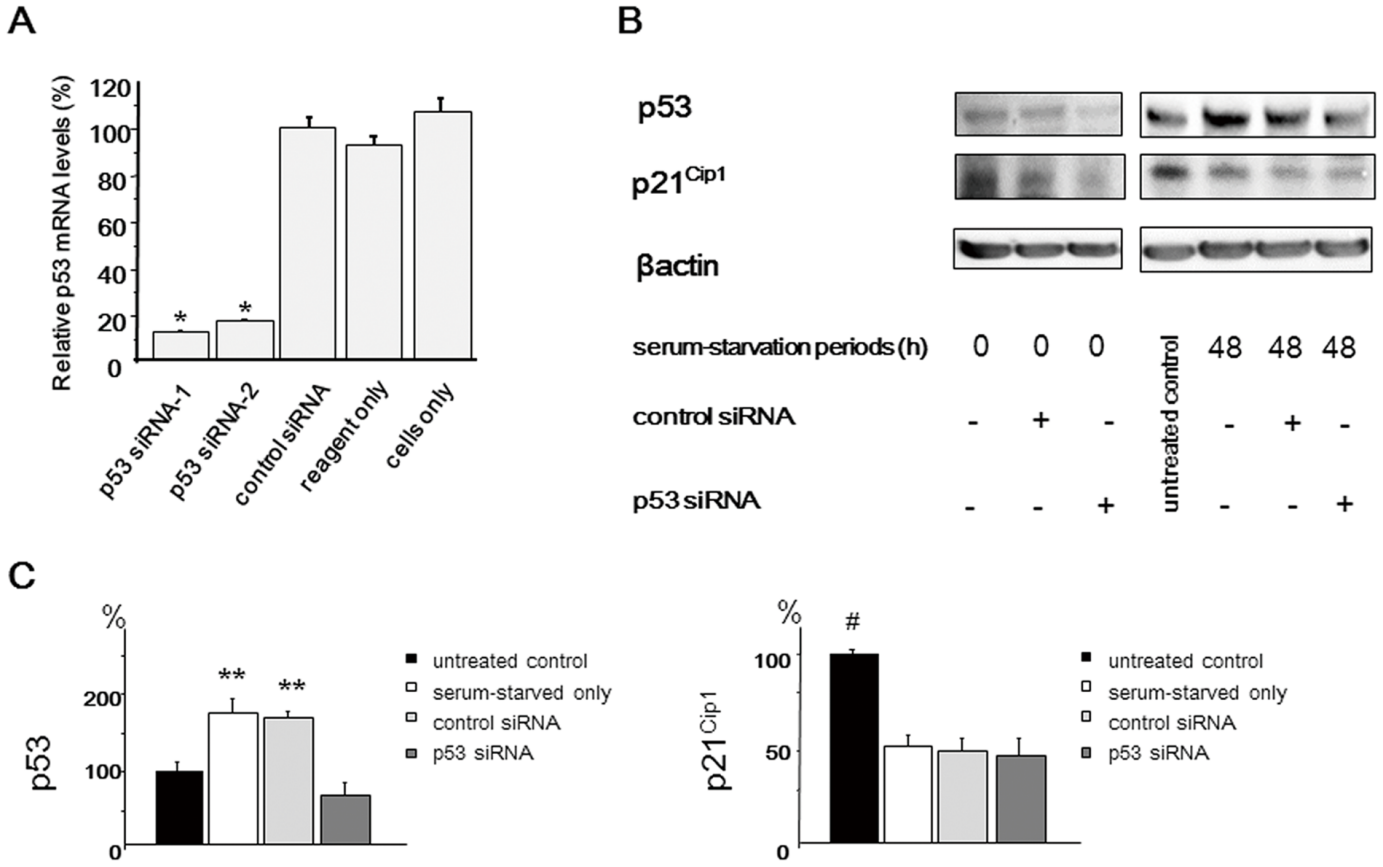
Functional interaction between p21^Cip1^ and p53 in serum-deprived rat nucleus pulposus (NP) cells. Forty-eight hours after p53 short interfering RNA (siRNA) transfection, cells were serum-deprived and harvested after 48 h. (**A**) qRT-PCR analysis of p53 mRNA expression was performed using rat NP cells transfected with p53 siRNA and a scrambled negative control siRNA. Total RNA was extracted 48 h after transfection, and glyceraldehyde phosphate dehydrogenase (GAPDH) expression was used for normalization. The results are expressed as a percentage of the expression in control siRNA-transfected cells. (**B**) Representative western blot analysis of protein extracts from NP cells. (**C**) Densitometry analyses were performed to quantify the levels of p53 and p21^Cip1^ 48 h after serum starvation via normalization to beta-actin. p21^Cip1^ expression was decreased in p53 siRNA-transfected cells before serum starvation (48 h after p53 siRNA transfection). However, there was no significant difference in the expression level of p21^Cip1^ among serum-starved only, control siRNA-transfected, and p53 siRNA-transfected cells at 48 h after serum starvation. Results are representative of three independent experiments. Values are expressed as the mean ± SD (* = P<0.05 compared with control siRNA, reagent only, and untreated cells, ** = P<0.05 compared with untreated control and p53 siRNA, # = P<0.05 versus all other groups).

Next, we examined the functional interaction between p21^Cip1^ and p53 in NP cells. The endogenous p53 mRNA levels in the NP cells significantly decreased after transfection with p53 siRNAs (**Figure 2A**). The sequence-1 p53 siRNA was selected for the following study. Western blot analysis demonstrated that p21^Cip1^ expression was decreased in p53 siRNA-transfected cells before serum starvation (48 h after p53 siRNA transfection). However, there was no significant difference in the expression level of p21^Cip1^ among serum-starved only, control siRNA-transfected, and p53 siRNA-transfected cells at 48 h after serum starvation. Thus, it is suggested that p21^Cip1^ is regulated independently of p53 under the serum-starved condition, although p53 influences on p21^Cip1^ expression under the nutrient condition. (**Figure 2**).

### Functional Interaction between p21^Cip1^ and Caspase 3 in Serum-deprived NP Cells

We next investigated the functional interaction between p21^Cip1^ and caspase 3 in NP cells. p21^Cip1^ inhibits cyclin-CDK complex activity as a CKI as well as regulates apoptosis by integrating procaspase 3 [18]–[20]. Conversely, it has been reported that caspase 3 cleaves and inactivates p21^Cip1^ when apoptotic pathways are strongly activated [21]. Furthermore, it was recently demonstrated that the gene silencing of caspase 3 was effective for blocking NP cell apoptosis and slowing IVD degeneration both *in vitro* and *in vivo* [8], [22]. Thus, it is important to investigate the functional interaction between p21^Cip1^ and caspase 3 in NP cells.

**Figure 3:**
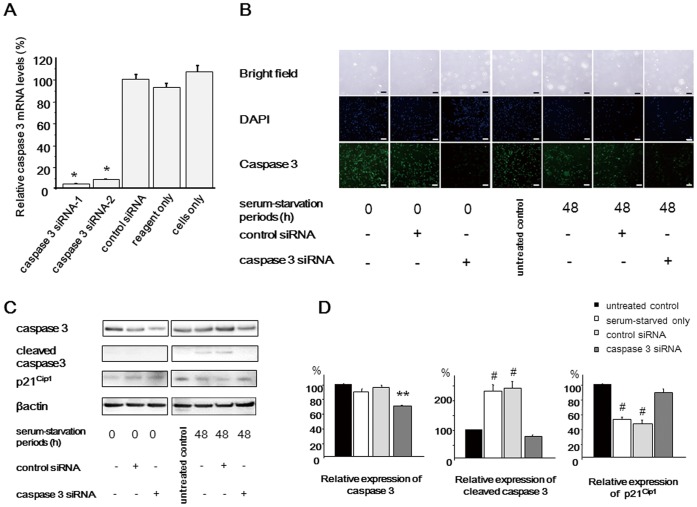
Functional interaction between p21^Cip1^ and caspase 3 in serum-deprived rat nucleus pulposus (NP) cells. Forty-eight hours after caspase 3 siRNA transfection, cells were serum-deprived (**A**) qRT-PCR analysis of caspase 3 mRNA expression was performed using rat NP cells transfected with caspase 3 siRNA and a scrambled negative control siRNA. Total RNA was extracted 48 h after transfection, and glyceraldehyde phosphate dehydrogenase (GAPDH) expression was used for normalization. The results are expressed as a percentage of the expression in control siRNA-transfected cells. (**B**) Caspase 3 expression was evaluated by immunofluorescence analysis. Caspase 3 expression decreased in cells transfected with caspase 3 siRNA. Bar = 200 µm (**C**) Representative western blot analysis of protein extracts from NP cells. (**D**) Densitometry analyses were performed to quantify the levels of caspase 3 and p21^Cip1^ 48 h after serum starvation via normalization to beta-actin. p21^Cip1^ protein expression remained in caspase 3 siRNA-transfected cells, indicating that caspase 3 mediates p21^Cip1^ cleavage in serum-deprived NP cells. Results are representative of three independent experiments. Values are expressed as the mean ± SD (* = P<0.05 compared with control siRNA, reagent only, and untreated cells, ** = P<0.05 compared with all other groups, # = P<0.05 versus untreated control and caspase 3 siRNA).

Caspase 3 siRNAs were constructed and transfected into NP cells. We initially confirmed the significant decrease of caspase 3 mRNA levels after transfection of caspase 3 siRNAs (**Figure 3A**). The sequence-1 caspase 3 siRNA was selected for the following study. After 48 h of serum starvation (96 h after siRNA transfection), rat NP cells were harvested for PCR array and western blot analysis. PCR array analysis revealed that p21^Cip1^ mRNA levels were significantly increased (**Table 3**). The other genes significantly regulated by caspase 3 are also shown in **Table 3**. Western blot analysis demonstrated that p21^Cip1^ remained in caspase 3 siRNA-transfected cells, indicating that caspase 3 mediates p21^Cip1^ cleavage in serum-deprived NP cells (**Figure 3**). Western bot analysis displayed the same expression trends as human NP cells (**Figure S3**).

**Table 3:** Genes with significant expression levels in serum-starved nucleus pulposus cells transfected with caspase 3 siRNA.

Gene symbol	Description	Fold change	*P* value
Casp3	Caspase 3, apoptosis related cysteine protease	−22.51	0.000038
Ccna2	Cyclin A2	−1.92	0.033772
Ccnb2	Cyclin B2	−2.01	0.000215
Cdkn1a	Cyclin-dependent kinase inhibitor 1A (p21^Cip1^)	1.61	0.000000
Cdkn1b	Cyclin-dependent kinase inhibitor 1B(p27^Kip1^)	1.20	0.022136
Chek1	CHK1 checkpoint homolog (S. pombe)	−1.26	0.009386
Ddit3	DNA-damage inducible transcript 3	−1.18	0.079602
Dnajc2	DnaJ (Hsp40) homolog, subfamily C, member 2	−1.05	0.002712
LOC289740	Similar to PES1 protein	−1.22	0.007056
E2f3	E2F transcription factor 3	−1.43	0.000482
Hus1	HUS1 checkpoint homolog (S. pombe)	−1.17	0.000981
Mad2l1	MAD2 (mitotic arrest deficient, homolog)-like 1 (yeast)	−1.67	0.000388
Mcm4	Minichromosome maintenance complex component 4	−1.32	0.021914
Npm2	Nucleophosmin/nucleoplasmin 2	−1.45	0.000564
Pkd1	Polycystic kidney disease 1 homolog (human)	1.34	0.012155
Pmp22	Peripheral myelin protein 22	−1.37	0.000479
Ran	RAN, member RAS oncogene family	−1.65	0.000005
Sfn	Stratifin	−1.29	0.046112
Taf10	TAF10 RNA polymerase II, TATA box binding protein-associated factor	1.43	0.000465

**Figure 4:**
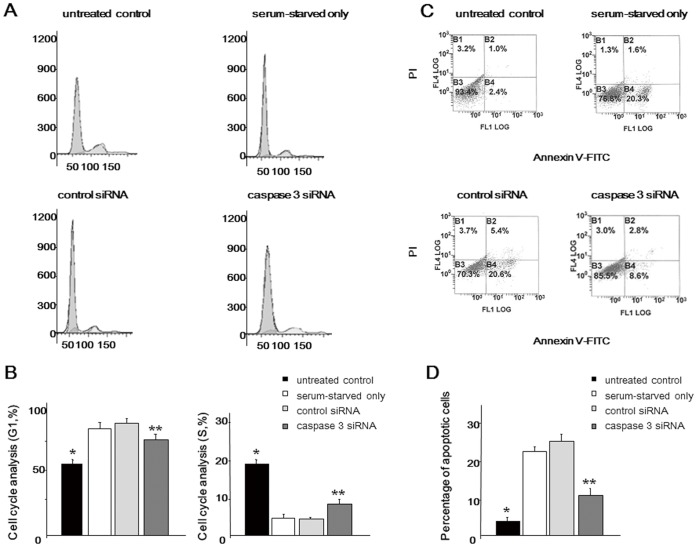
Flow cytometric analysis of rat nucleus pulposus (NP) cells. Forty-eight hours after caspase 3 siRNA transfection, cells were serum-deprived and harvested after 48 h. (**A**) Representative graphs showing the cell cycle. (**B**) Comparison of the cell cycle in the G1 and S phase. (**C**) The dual parametric dot plots combining annexin V-FITC and PI fluorescence show the early apoptotic cells (FITC+/PI -), and the late apoptotic cells (FITC+/PI +). (**D**) Percentage of (early+late) apoptotic cells. Results are representative of three independent experiments. Values are expressed as the mean ± SD (* = P<0.05 versus all other groups, ** = P<0.05 versus serum-starved only and control siRNA).

Further functional tests using siRNA were performed to verify that cell cycle/apoptosis regulation is essential for maintenance of the biological activity of NP cells. The results demonstrated that serum starvation induced significant G1 arrest and apoptotic alterations. However, both G1 arrest and apoptosis were significantly inhibited in the caspase 3 siRNA group compared with serum-starved only and control siRNA groups, indicating that the rescue of the cell phenotype is important for maintaining the biological activity of NP cells under a nutrient-deprived condition (**Figure 4**). Flow cytometric analysis displayed the same trends as human NP cells (**Figure S4**).

## Discussion

NP has an avascular structure encapsulated by an endplate and the annulus fibrosus. Moreover, in degenerated IVD tissue, the nutrient supply is compromised by endplate consolidation. Although an adequate cell population is needed in the disc to maintain normal homeostasis and restoration of cell numbers would be an important requirement for reversing the process of degeneration [5], [23], there have been a few researches about significant differences in deteriorated IVDs. Rannou et al. reported an association of human IVD degeneration with disc cell apoptosis [24]. They revealed that the activation of the mitochondria-dependent apoptosome was a major event in intervertebral degeneration. Gruber et al. used a laser capture microdissection technique to harvest human annuli and derived gene expression profiles using microarray analysis [25], [26]. They identified that genes with biological significance were regulated during degeneration involving cell senescence and low cell division rates.

We focused on NP cells derived from the IVD because cells constituting the NP are known to be sparse and have low self-renewal capacity. It is important to understand the gene expression profiles in nutrient-deprived NP cells. Nutrient deprivation reportedly induces NP cell apoptosis and decreases the expression of type II collagen and aggrecan, two basic genes important for NP homeostasis [7], [14]. In addition, we previously demonstrated that preventing NP cell apoptosis results in significantly higher levels of type II collagen and aggrecan [7]. On the basis of these previous NP-specific results, we performed the present study using the same cells and experimental design. According to the present results based on GO and KEGG pathway analysis, categories related to *response to nutrient levels* and *regulation of cell proliferation and cell cycle* were significantly altered, which demonstrated that this *in vitro* serum starvation model was appropriate for simulating IVD degeneration via nutrient deprivation.

As microarray data validation should be performed as a first step in subsequent studies of the regulation or function of specific genes, we subsequently focused on cell cycle-related pathways and validated which cell cycle-related genes were involved using PCR arrays. Among cell cycle-related factors, we observed increased expression levels of several genes which are components of the DNA damage checkpoints in the human cell cycle [27]. The damage signal is detected by sensors and then introduced into effector molecules through mediators that participate in inhibiting cell cycle progression. In this study, *Atm*, *Hus1*, *Rad9*, and *Rad17* were identified as sensor genes, *Brca1* was identified as a mediator, and *Cdc25* and *Tp53* were identified as effector genes. *Tp53*, a known tumor suppressor gene, regulates the transcriptional levels of genes associated with cell cycle arrest, DNA repair, and apoptosis. The mRNA levels of *Gadd45* were also increased in serum-starved NP cells. GADD45 is a DNA repair-associated molecule and is one of the targets of p53 [28]. GADD45 proteins control the balance between DNA repair and apoptosis [29]. Ijiri et al. reported that GADD45β was expressed at higher levels in cartilage from patients with early osteoarthritis (OA) than in cartilage from patients with late-stage OA, and suggested that GADD 45β plays an important role in regulating chondrocyte homeostasis by promoting cell survival in early OA [30].

Both p21^Cip1^ and p27^Kip1^ are members of the Cip/Kip family of CKIs. p21^Cip1^ is also one of the representative target of p53 activated by DNA damage, and it is deeply involved in cellular senescence and cell cycle arrest [31]. This cell cycle arrest is considered necessary for permitting DNA damage repair. The present study revealed that p21^Cip1^ was increased in the early phase of serum starvation (6 h) but decreased after 48 h of starvation. Mammalian cells respond to DNA damage signals by activating cell cycle checkpoints that arrest the cell cycle or by inducing apoptotic cell death. These results indicate that the increased expression of p21^Cip1^ in the early phase of serum starvation is probably a protective mechanism aimed at coping with the stress of nutrient deprivation, but it tends to participate in apoptosis when DNA repair is required.

Nakai et al. demonstrated that transforming growth factor beta 1 promoted proliferation and cell cycle progression while reducing the expression of p21^Cip1^ and p27^kip1^ in rat NP cells [32]. They also reported that no distinguishable change was observed in p15^Ink4b^ expression. p15^Ink4b^ belongs to another CKI family, the INK4 family. In this study, the expression of both p15^Ink4b^ and p16^Ink4a^ was not changed in microarray and PCR array analyses (data not shown). In addition, no change in their expression was identified in NP cells by western blotting even after 48 h of serum starvation. These results suggest that the Cip/Kip family of CKIs plays important roles in the NP cell cycle. However, Le Maitre et al. documented that cells from degenerated human IVDs exhibited increased expression of the biomarker of cell senescence p16^Ink4a^ [5]. Conversely, Dai et al. reported that both interleukin-1 beta and oxidative stress upregulated p53 and p21^Cip1^ but did not induce p16^Ink4a^ in chondrocytes [33]. Further investigations should be conducted to clarify the interactive mechanism among proliferation, cell cycle, senescence, and apoptosis of IVD cells.

In this study, p21^Cip1^ remained in caspase 3 siRNA-transfected cells. It has been reported that p21^Cip1^ is specifically cleaved by caspase 3, which is instrumental in the execution of apoptosis, and it contributes to the suppression of apoptosis [18]–[20]. In addition, the present study indicated that the rescue of the cell phenotype by caspase 3 gene silencing is important for maintaining the biological activity of NP cells under a nutrient-deprived condition. These findings indicated that p21^Cip1^ may improve IVD cell survival after caspase 3 gene silencing.

This study has some limitations. In the present study, an *in vitro* serum starvation model was selected to simulate nutrient deprivation in NP cells. However, complete serum starvation is an oversimplification of decreased nutrient delivery to NP in IVD degeneration. A more appropriate model would include physiologically and pathologically relevant nutrient levels. These would provide differentially expressed genes pertinent to the pathology of IVD degeneration. Another important component to be considered while conducting this study is its hypoxic nature. To closely mimic IVD, it would be important to conduct experiments with this in mind. These results could be compared with those of another cell type to determine whether these are NP-specific or global effects of serum starvation. It has been reported that under hypoxic conditions, rat NP cells are resistant to apoptosis induced by serum starvation [14]. In addition, we analyzed the effect of hypoxia (2% O_2_) on the protein expression of genes affected by serum depletion, such as p21^Cip1^, p27^Kip1^, p53, and caspase 3. The expression trends of these genes were identical to those under normoxia (20% O_2_); however, their expression levels were low compared with those under normoxia (**Figure S5**). Future experiments are needed to be determined what extent of hypoxia participate in nutrient deficiency in NP cell. Finally, this study did not investigate differentially expressed genes involved in proteoglycan and matrix synthesis. Changes in matrix composition during degeneration are well characterized. The present results showed that the O-glycan biosynthesis KEGG pathway had all downregulated elements in serum starvation; therefor, this would be an important pathway for further investigation.

In conclusion, this study provides new information about the genome-wide molecular response to nutrient deprivation. Nutrient deprivation in NP cells results in the activation of a signaling response that includes DNA damage checkpoint genes. These results provide a basis for further studies on the function of identified genes and their effects on IVD degeneration.

## Supporting Information

Figure S1 **Immunohistochemical staining of rat nucleus pulposus (NP) cells for type I collagen, type II collagen, and aggrecan.** For histologic detection of type I collagen, type II collagen, and aggrecan, immnunohistochemistry was performed. After the paraffin section were deparaffinized and rehydrated, sections were heated in a microwave for 5 min in 0.01 M citrate buffer (pH 6; collagen I and aggrecan) or treated with proteinase K for 6 min (collagen II). After washing with PBS, sections were treated with 1% H_2_O_2_-methanol for 30 min and incubated with type I collagen (1∶200;Abcam, UK), type II collagen (1∶50; Daiichi Fine Chemincal, Japan), and aggrecan (1∶100; Abcam) at room temperature for 60 minutes. The sections were then exposed to a peroxidase kit (EnVision+ System; Dako Japan), and color was developed with 3, 3′-diaminobenzidine hydrochloride (Dako Japan). Mayer’s hematoxylin was used for counterstaining. Type II collagen- and aggrecan-positive NP cells were apparent, indicating that *in vitro*-cultured NP cells were really cells with a feature of NP cells *in vivo*.
(TIF)

Figure S2 **Western blots of p15^Ink4b^, p16^Ink4a^, p21^Cip1^, and p27^Kip1^, and p53 in human nucleus pulposus cells.** Cells were harvested after 6 or 48 h of serum starvation. Cells not subjected to serum starvation were used as untreated controls. β-actin was used as an internal control. The results shown are representative of three independent experiments.
(TIF)

Figure S3 **Western blots of p21^Cip1^ and caspase 3 in human nucleus pulposus (NP) cells.** Forty-eight hours after caspase 3 siRNA transfection, cells were serum-deprived. (**A**) qRT-PCR analysis of caspase 3 mRNA expression was performed using NP cells transfected with caspase 3 siRNA and a scrambled negative control siRNA. Total RNA was extracted 48 h after transfection, and glyceraldehyde phosphate dehydrogenase (GAPDH) expression was used for normalization. The results are expressed as a percentage of the expression in control siRNA-transfected cells. The sequence-1 caspase 3 siRNA (caspase 3 siRNA-1) was selected for the following study. (**B**) Representative western blot analysis of protein extracts from NP cells. (**C**) Densitometry analyses were performed to quantify the levels of caspase 3 and p21^Cip1^ 48 h after serum starvation via normalization to beta-actin. p21^Cip1^ protein expression remained in caspase 3 siRNA-transfected cells, indicating that caspase 3 mediates p21^Cip1^ cleavage in serum-deprived NP cells. Results are representative of three independent experiments. Values are expressed as the mean ± SD (* = P<0.05 compared with control siRNA, reagent only, and untreated cells, ** = P<0.05 versus all other groups, # = P<0.05 versus untreated control and caspase 3 siRNA).
(TIF)

Figure S4 **Western blot analysis in serum-starved nucleus pulposus (NP) cells under hypoxia (2% O_2_).** Rat NP cells were harvested after 6 or 48 h of serum starvation under hypoxic conditions (2% O_2_). Cells not subjected to serum starvation were used as untreated controls. The results are representative of three independent experiments. Please refer to the result under normoxia (20% O_2_) in **Figure 1**.
(TIF)

Table S1 **Gene ontology (GO) terms in the biological process up-regulated by serum starvation.** Top 50 GO annotations with low *P* values were showed. “count” means the number of genes, which were expressed significantly in each pathway in this study. See precise description in the text.
(DOC)

Table S2 **Gene ontology (GO) terms in the biological process down-regulated by serum starvation.** Top 50 GO annotations with low *P* values were showed. “count” means the number of genes, which were expressed significantly in each pathway in this study. See precise description in the text.
(DOC)

Table S3 **List of up-regulated genes related to** ***response to nutrient levels*** **on gene ontology analysis.** Genes those are up-regulated in serum-starved nucleus pulposus cells as compared with control cells. The values for fold change are the mean from 3 independent experiments.
(DOC)
