# Supplementary figures and images for: Global Identification of Genes Related to Nutrient Deficiency in Intervertebral Disc Cells in an Experimental Nutrient Deprivation Model
Source: PLoS One. 2013 Mar 8;8(3):e58806. doi: 10.1371/journal.pone.0058806 (PMC3592817; doi:10.1371/journal.pone.0058806)

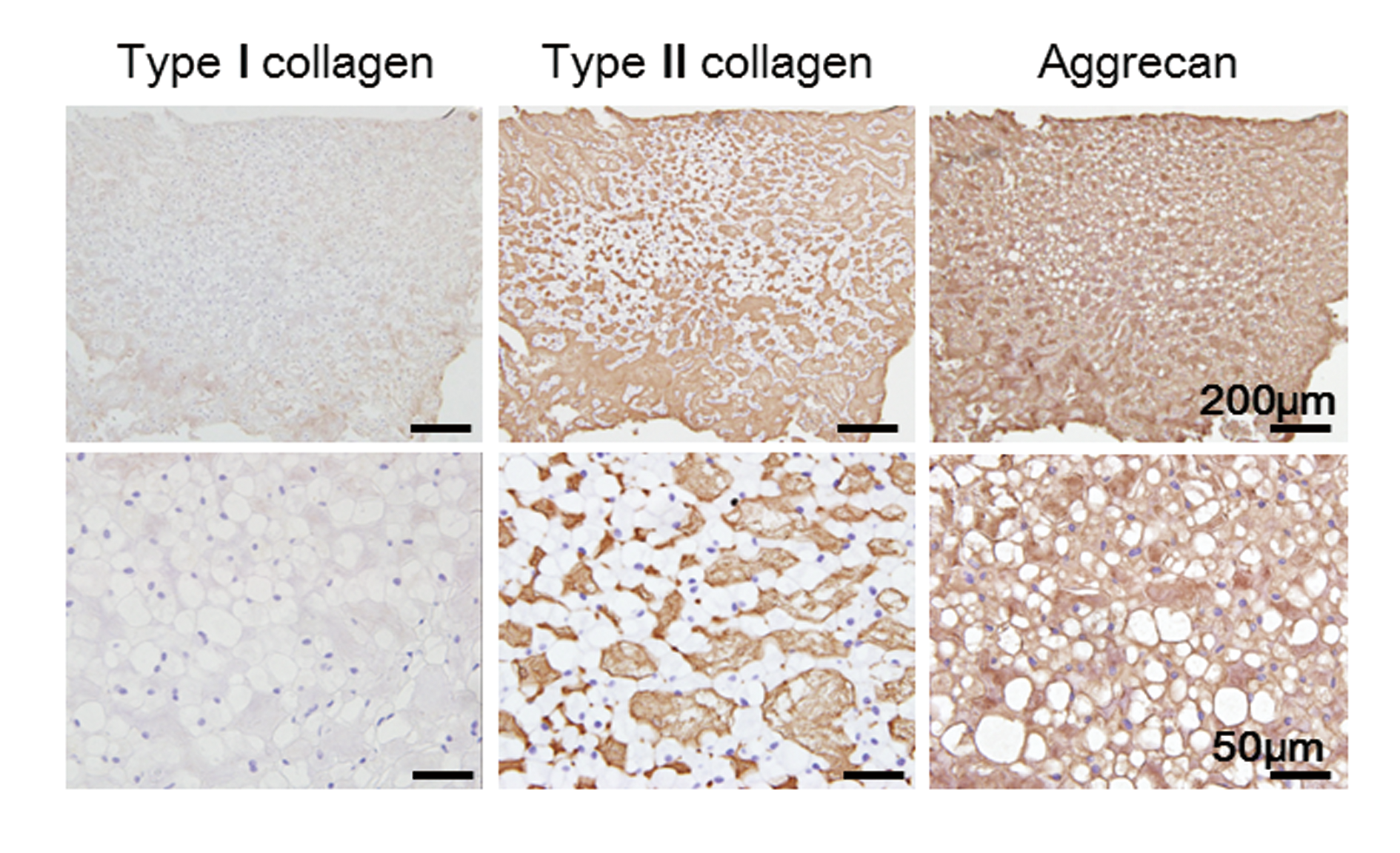

Supplement: Figure S1 — Immunohistochemical staining of rat nucleus pulposus (NP) cells for type I collagen, type II collagen, and aggrecan. For histologic detection of type I collagen, type II collagen, and aggrecan, immnunohistochemistry was performed. After the paraffin section were deparaffinized and rehydrated, sections were heated in a microwave for 5 min in 0.01 M citrate buffer (pH 6; collagen I and aggrecan) or treated with proteinase K for 6 min (collagen II). After washing with PBS, sections were treated with 1% H2O2-methanol for 30 min and incubated with type I collagen (1∶200;Abcam, UK), type II collagen (1∶50; Daiichi Fine Chemincal, Japan), and aggrecan (1∶100; Abcam) at room temperature for 60 minutes. The sections were then exposed to a peroxidase kit (EnVision+ System; Dako Japan), and color was developed with 3, 3′-diaminobenzidine hydrochloride (Dako Japan). Mayer’s hematoxylin was used for counterstaining. Type II collagen- and aggrecan-positive NP cells were apparent, indicating that in vitro-cultured NP cells were really cells with a feature of NP cells in vivo. (TIF) [file pone.0058806.s001.tif]

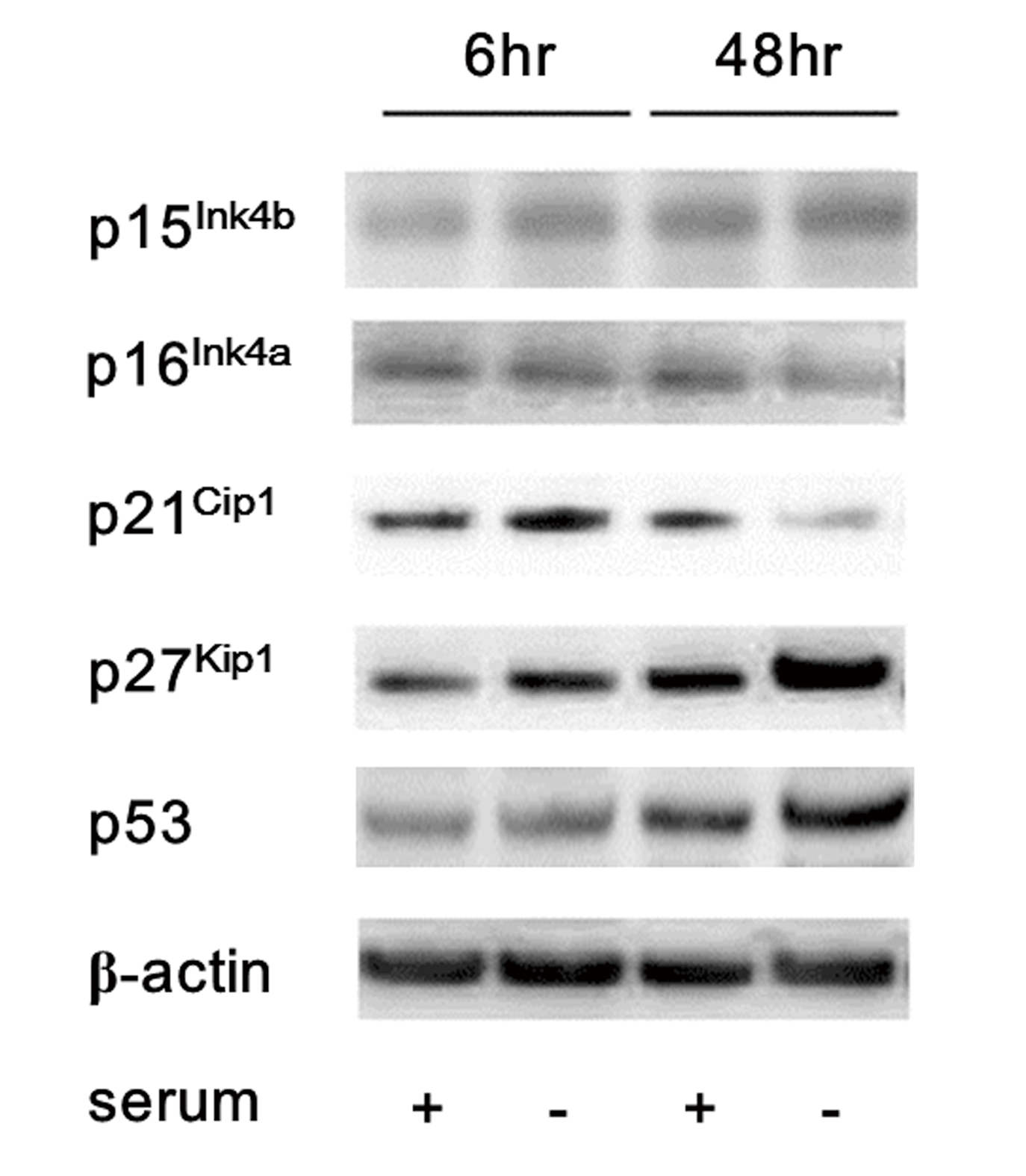

Supplement: Figure S2 — Western blots of p15Ink4b, p16Ink4a, p21Cip1, and p27Kip1, and p53 in human nucleus pulposus cells. Cells were harvested after 6 or 48 h of serum starvation. Cells not subjected to serum starvation were used as untreated controls. β-actin was used as an internal control. The results shown are representative of three independent experiments. (TIF) [file pone.0058806.s002.tif]

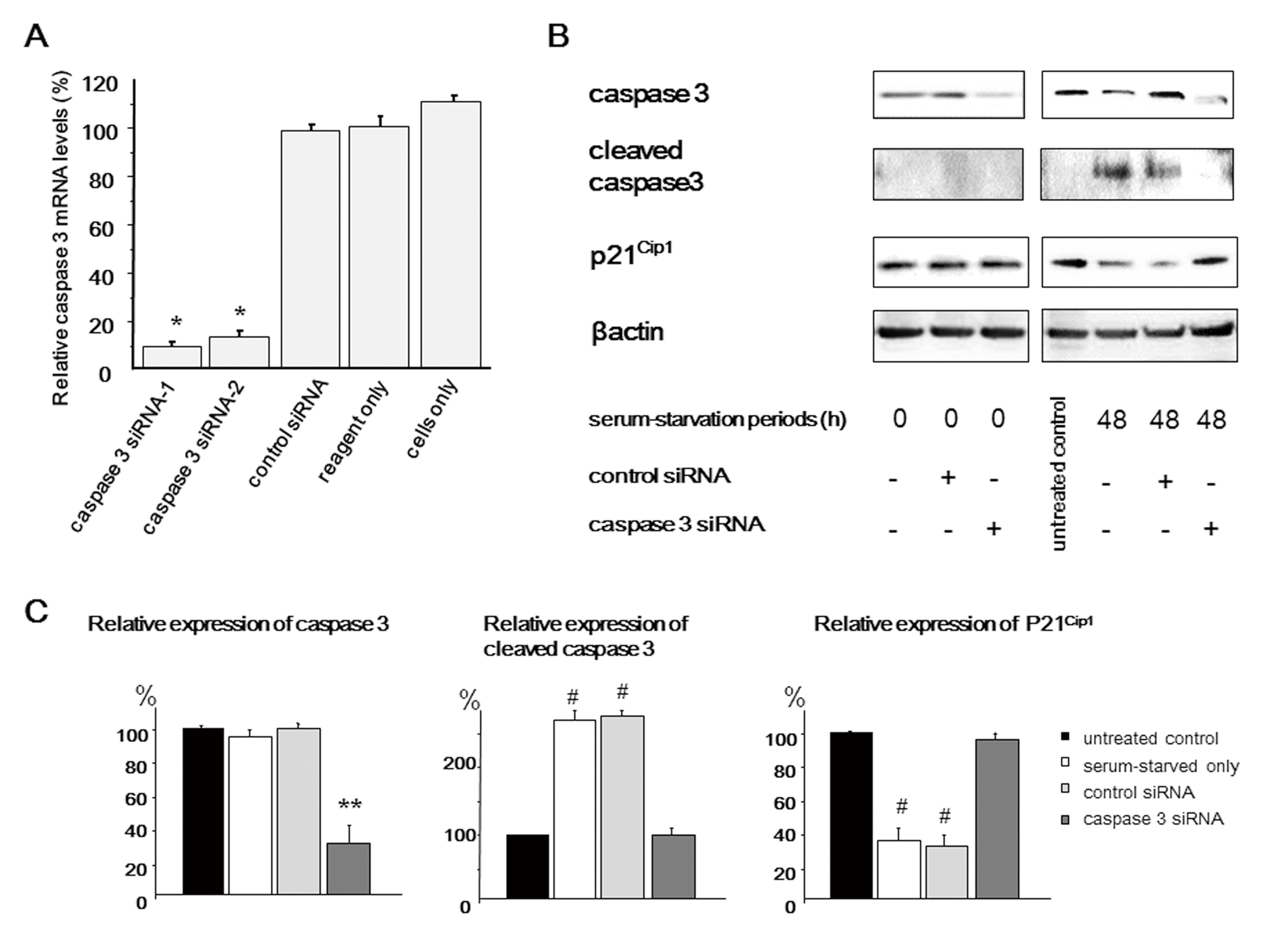

Supplement: Figure S3 — Western blots of p21Cip1 and caspase 3 in human nucleus pulposus (NP) cells. Forty-eight hours after caspase 3 siRNA transfection, cells were serum-deprived. (A) qRT-PCR analysis of caspase 3 mRNA expression was performed using NP cells transfected with caspase 3 siRNA and a scrambled negative control siRNA. Total RNA was extracted 48 h after transfection, and glyceraldehyde phosphate dehydrogenase (GAPDH) expression was used for normalization. The results are expressed as a percentage of the expression in control siRNA-transfected cells. The sequence-1 caspase 3 siRNA (caspase 3 siRNA-1) was selected for the following study. (B) Representative western blot analysis of protein extracts from NP cells. (C) Densitometry analyses were performed to quantify the levels of caspase 3 and p21Cip1 48 h after serum starvation via normalization to beta-actin. p21Cip1 protein expression remained in caspase 3 siRNA-transfected cells, indicating that caspase 3 mediates p21Cip1 cleavage in serum-deprived NP cells. Results are representative of three independent experiments. Values are expressed as the mean ± SD (* = P<0.05 compared with control siRNA, reagent only, and untreated cells, ** = P<0.05 versus all other groups, # = P<0.05 versus untreated control and caspase 3 siRNA). (TIF) [file pone.0058806.s003.tif]

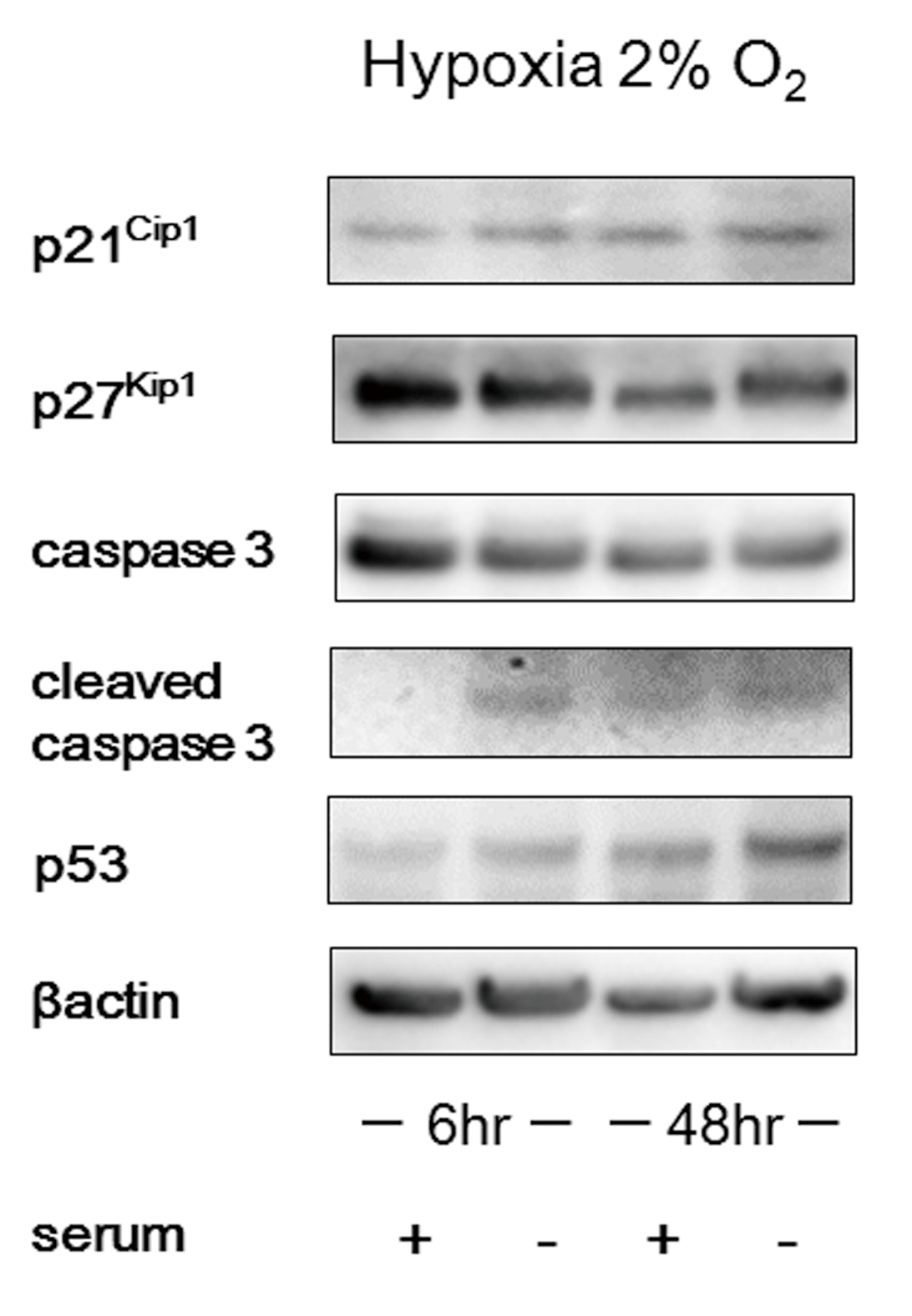

Supplement: Figure S4 — Western blot analysis in serum-starved nucleus pulposus (NP) cells under hypoxia (2% O2). Rat NP cells were harvested after 6 or 48 h of serum starvation under hypoxic conditions (2% O2). Cells not subjected to serum starvation were used as untreated controls. The results are representative of three independent experiments. Please refer to the result under normoxia (20% O2) in Figure 1 . (TIF) [file pone.0058806.s004.tif]
