# Supplementary material for: Global Identification of Genes Related to Nutrient Deficiency in Intervertebral Disc Cells in an Experimental Nutrient Deprivation Model
Source: PLoS One. 2013 Mar 8;8(3):e58806. doi: 10.1371/journal.pone.0058806 (PMC3592817; doi:10.1371/journal.pone.0058806)
Supplement: Table S1 — Gene ontology (GO) terms in the biological process up-regulated by serum starvation. Top 50 GO annotations with low P values were showed. “count” means the number of genes, which were expressed significantly in each pathway in this study. See precise description in the text. (DOC) [file pone.0058806.s005.doc]

**Table S1.** Gene ontology (GO) terms in the biological process

up-regulated by serum starvation*

| GO annotation | *P*-value | Count |
| --- | --- | --- |
| response to external stimulus | 6.43 x 10^-13^ | 64 |
| regulation of biological process | 4.82 x 10^-12^ | 191 |
| regulation of cellular process | 1.66 x 10^-11^ | 170 |
| developmental process | 1.02 x 10^-10^ | 155 |
| biological regulation | 2.2 x 10^-10^ | 200 |
| response to wounding | 6.09 x 10^-10^ | 42 |
| anatomical structure development | 8.89 x 10^-10^ | 122 |
| response to stress | 9.36 x 10^-10^ | 75 |
| immune system process | 1.67 x 10^-09^ | 59 |
| system development | 5.54 x 10^-09^ | 110 |
| positive regulation of biological process | 6.73 x 10^-09^ | 90 |
| multicellular organismal development | 2.42 x 10^-08^ | 118 |
| positive regulation of cellular process | 3.82 x 10^-08^ | 81 |
| cellular developmental process | 1.66 x 10^-07^ | 104 |
| cell differentiation | 1.66 x 10^-07^ | 104 |
| inflammatory response | 1.76 x 10^-07^ | 26 |
| Behavior | 1.94 x 10^-07^ | 35 |
| response to extracellular stimulus | 2.89 x 10^-07^ | 21 |
| organ development | 4.69 x 10^-07^ | 88 |
| defense response | 7.88 x 10^-07^ | 35 |
| lipid metabolic process | 9.14 x 10^-07^ | 47 |
| response to nutrient levels | 9.56 x 10^-07^ | 20 |
| regulation of cell proliferation | 1.20 x 10^-06^ | 43 |
| immune response | 1.91 x 10^-06^ | 36 |
| regulation of multicellular organismal process | 2.02 x 10^-06^ | 39 |
| regulation of developmental process | 4.59 x 10^-06^ | 34 |
| locomotory behavior | 4.78 x 10^-06^ | 21 |
| cellular lipid metabolic process | 7.26 x 10^-06^ | 41 |
| cell development | 7.41 x 10^-06^ | 79 |
| regulation of metabolic process | 8.11 x 10^-06^ | 102 |
| tissue development | 1.27 x 10^-05^ | 31 |
| cellular component organization and biogenesis | 1.42 x 10^-05^ | 118 |
| regulation of cellular metabolic process | 2.28 x 10^-05^ | 94 |
| membrane organization and biogenesis | 2.46 x 10^-05^ | 24 |
| carboxylic acid metabolic process | 3.15 x 10^-05^ | 40 |
| organic acid metabolic process | 3.15 x 10^-05^ | 40 |
| positive regulation of apoptosis | 4.63 x 10^-05^ | 25 |
| positive regulation of programmed cell death | 5.13 x 10^-05^ | 25 |
| anatomical structure morphogenesis | 5.77 x 10^-05^ | 68 |
| cell proliferation | 8.17 x 10^-05^ | 46 |
| negative regulation of cellular process | 8.55 x 10^-05^ | 65 |
| regulation of programmed cell death | 8.97 x 10^-05^ | 41 |
| regulation of cell differentiation | 0.000112264 | 24 |
| response to other organism | 0.000117083 | 20 |
| response to biotic stimulus | 0.00012937 | 23 |
| response to drug | 0.00014258 | 24 |
| nervous system development | 0.000146085 | 49 |
| response to organic substance | 0.000171546 | 26 |
| negative regulation of biological process | 0.000174563 | 68 |
| regulation of apoptosis | 0.00028338 | 39 |

^*^Top 50 GO annotations with low *P* values were showed. “count”

means the number of genes, which were expressed significantly in

each pathway in this study. See precise description in the text.
