# Supplementary material for: Global Identification of Genes Related to Nutrient Deficiency in Intervertebral Disc Cells in an Experimental Nutrient Deprivation Model
Source: PLoS One. 2013 Mar 8;8(3):e58806. doi: 10.1371/journal.pone.0058806 (PMC3592817; doi:10.1371/journal.pone.0058806)
Supplement: Table S2 — Gene ontology (GO) terms in the biological process down-regulated by serum starvation. Top 50 GO annotations with low P values were showed. “count” means the number of genes, which were expressed significantly in each pathway in this study. See precise description in the text. (DOC) [file pone.0058806.s006.doc]

**Table S2.** Gene ontology (GO) terms in the biological process

down-regulated by serum starvation^*^

^*^Top 50 GO annotations with low *P* values were showed. “count”

| GO annotation | *P*-value | Count |
| --- | --- | --- |
| anatomical structure development | 2.17 x 10^-19^ | 147 |
| developmental process | 3.35 x 10^-19^ | 178 |
| system development | 1.44 x 10^-18^ | 135 |
| multicellular organismal development | 3.78 x 10^-18^ | 145 |
| organ development | 1.64 x 10^-15^ | 111 |
| anatomical structure morphogenesis | 1.11 x 10^-11^ | 88 |
| cell development | 3.77 x 10^-11^ | 95 |
| response to external stimulus | 4.06 x 10^-11^ | 60 |
| response to wounding | 4.9 x 10^-10^ | 42 |
| cellular developmental process | 8.63 x 10^-10^ | 111 |
| cell differentiation | 8.63 x 10^-10^ | 111 |
| multicellular organismal process | 9.86 x 10^-10^ | 196 |
| regulation of cell proliferation | 2.06 x 10^-08^ | 47 |
| nervous system development | 2.39 x 10^-08^ | 60 |
| organ morphogenesis | 2.91 x 10^-08^ | 48 |
| cell proliferation | 4.72 x 10^-08^ | 55 |
| tissue development | 1.74 x 10^-07^ | 35 |
| negative regulation of biological process | 2.35 x 10^-07^ | 78 |
| positive regulation of biological process | 3.73 x 10^-07^ | 84 |
| growth | 6.44 x 10^-07^ | 34 |
| regulation of multicellular organismal process | 6.53 x 10^-07^ | 40 |
| anatomical structure formation | 8.84 x 10^-07^ | 24 |
| response to stress | 1.03 x 10^-06^ | 66 |
| positive regulation of cell proliferation | 1.15 x 10^-06^ | 31 |
| wound healing | 1.30 x 10^-06^ | 21 |
| neurogenesis | 1.38 x 10^-06^ | 40 |
| blood vessel development | 1.38 x 10^-06^ | 24 |
| blood vessel morphogenesis | 1.47 x 10^-06^ | 22 |
| regulation of biological process | 1.48 x 10^-06^ | 169 |
| cell death | 1.63 x 10^-06^ | 54 |
| vasculature development | 1.84 x 10^-06^ | 24 |
| heart development | 1.84 x 10^-06^ | 20 |
| death | 1.94 x 10^-06^ | 54 |
| apoptosis | 2.15 x 10^-06^ | 52 |
| biological regulation | 2.8 x 10^-06^ | 182 |
| regulation of biological quality | 3.01 x 10^-06^ | 51 |
| programmed cell death | 3.07 x 10^-06^ | 52 |
| regulation of programmed cell death | 3.19 x 10^-06^ | 45 |
| circulation | 3.89 x 10^-06^ | 20 |
| regulation of developmental process | 3.93 x 10^-06^ | 34 |
| second-messenger-mediated signaling | 4.28 x 10^-06^ | 21 |
| neuron differentiation | 4.33 x 10^-06^ | 34 |
| regulation of apoptosis | 5.22 x 10^-06^ | 44 |
| generation of neurons | 5.49 x 10^-06^ | 36 |
| negative regulation of cellular process | 5.54 x 10^-06^ | 69 |
| biological adhesion | 6.06 x 10^-06^ | 37 |
| cell adhesion | 6.06 x 10^-06^ | 37 |
| positive regulation of cellular process | 1.44 x 10^-05^ | 72 |
| cell migration | 1.64 x 10^-05^ | 30 |
| cell morphogenesis | 1.66 x 10^-05^ | 40 |

means the number of genes, which were expressed significantly in

each pathway in this study. See precise description in the text.
