# Supplementary material for: Global Identification of Genes Related to Nutrient Deficiency in Intervertebral Disc Cells in an Experimental Nutrient Deprivation Model
Source: PLoS One. 2013 Mar 8;8(3):e58806. doi: 10.1371/journal.pone.0058806 (PMC3592817; doi:10.1371/journal.pone.0058806)
Supplement: Table S3 — List of up-regulated genes related to response to nutrient levels on gene ontology analysis. Genes those are up-regulated in serum-starved nucleus pulposus cells as compared with control cells. The values for fold change are the mean from 3 independent experiments. (DOC) [file pone.0058806.s007.doc]

**Table S3.** List of up-regulated genes related to *response to nutrient levels* on gene ontology analysis.^*^

^*^Genes those are up-regulated in serum-starved nucleus pulposus cells as compared with control cells. The values for fold change are the mean from 3 independent experiments.

| Gene Name | Entrez Gene No | Fold Change |
| --- | --- | --- |
| Apolipoprotein C-III | 24207 | 2.95 |
| B-cell leukemia/lymphoma 2 | 24224 | 1.82 |
| Glucokinase | 24385 | 1.85 |
| Jun oncogene | 24516 | 1.65 |
| Interleukin 6 signal transducer | 25205 | 1.63 |
| Oxytocin | 25504 | 2.72 |
| Glucose-6-phosphatase, catalytic | 25634 | 1.58 |
| Microtubule-associated protein tau | 29477 | 1.53 |
| Solute carrier family 8, member 1 | 29715 | 2.48 |
| Transforming growth factor, beta receptor II | 81810 | 1.64 |
| Harakiri, BCL2 interacting protein | 117271 | 6.29 |
| NUAK family, SNF1-like kinase, 2 | 289419 | 1.59 |
| Mucin 4 | 303887 | 3.92 |
| ATP-binding cassette, sub-family A, member 1 | 313210 | 3.40 |
| Breast cancer 2 | 360254 | 1.69 |
